# Prediction of Sleep Apnea Events Using a CNN–Transformer Network and Contactless Breathing Vibration Signals

**DOI:** 10.3390/bioengineering10070746

**Published:** 2023-06-21

**Authors:** Yuhang Chen, Shuchen Yang, Huan Li, Lirong Wang, Bidou Wang

**Affiliations:** 1 School of Biomedical Engineering (Suzhou), Division of Life Sciences and Medicine, University of Science and Technology of China, Hefei 230026, China; 2 Suzhou Institute of Biomedical Engineering and Technology, Chinese Academy of Sciences, Suzhou 215163, China; 3 Shanghai Yueyang Medtech Co., Shanghai 200131, China; 4 Department of Sleep Medical Center, Beijing Anzhen Hospital, Capital Medical University, No. 2 Anzhen Road, Beijing 100029, China; 5 Department of Ultrasound, Xinhua Hospital Affiliated to Shanghai Jiaotong University School of Medicine, Shanghai 200092, China; 6 School of Electronics and Information Technology, Soochow University, Suzhou 215006, China

**Keywords:** respiratory event prediction, transformer, CNN, contactless monitoring

## Abstract

It is estimated that globally 425 million subjects have moderate to severe obstructive sleep apnea (OSA). The accurate prediction of sleep apnea events can offer insight into the development of treatment therapies. However, research related to this prediction is currently limited. We developed a covert framework for the prediction of sleep apnea events based on low-frequency breathing-induced vibrations obtained from piezoelectric sensors. A CNN-transformer network was utilized to efficiently extract local and global features from respiratory vibration signals for accurate prediction. Our study involved overnight recordings of 105 subjects. In five-fold cross-validation, we achieved an accuracy of 85.9% and an F1 score of 85.8%, which are 3.5% and 5.3% higher than the best-performed classical model, respectively. Additionally, in leave-one-out cross-validation, 2.3% and 3.8% improvements are observed, respectively. Our proposed CNN-transformer model is effective in the prediction of sleep apnea events. Our framework can thus provide a new perspective for improving OSA treatment modes and clinical management.

## 1. Introduction

Obstructive sleep apnea (OSA) is a highly prevalent disorder characterized by the instability of the upper airway during sleep, which results in markedly reduced (hypopnea) or absent (apnea) airflow at the nose/mouth, leading to disrupted sleep and drops in blood oxygen levels [1]. OSA can have substantial consequences in the long term, such as hypertension and cardiovascular morbidities [2,3,4]. According to a systematic review, the prevalence of OSA in the average adult, with an Apnea–Hypopnea Index (AHI) of ≥15 events/h, ranges from 6% to 17%. The prevalence can be as high as 49% in advanced ages [5]. Another study estimated that 936 million adults aged 30–69 years have mild to severe OSA [6].

Positive airway pressure (PAP) is the primary treatment for OSA. It provides a positive airflow to the upper airway to prevent airway collapse and obstruction, thereby improving respiratory events, sleep quality, and quality of life [7]. To improve treatment compliance and effectiveness, in addition to the original continuous PAP (CPAP), more PAP therapy modes have been developed, including automatic titration CPAP and bi-level PAP. A recent study showed that positional OSA was present in 53% of the general population and in 75% of OSA subjects [8]. Positional therapy can diminish OSA severity in positional OSA patients. Thus, some studies have focused on inducing head and posture changes with a pillow [9,10]. The prediction of sleep apnea events may provide strategies for developing PAP therapy modes and pillow adjustment algorithms.

Many studies have been conducted in sleep apnea detection. Pant et al. [11] proposed an ECG-based sleep apnea detection method using flexible analytic wavelet transform and optimize ensemble classifier. Nassi et al. [12] developed a neural network approach based on WaveNet and a respiratory effort signal from a single belt to screen for sleep apnea. Chen et al. [13] proposed a lightweight multi-scaled neural network for sleep apnea detection based on single-lead ECG signals. Meanwhile, few methods and algorithms have been proposed to predict sleep apnea events using multiple physiological signals. The study in Waxman et al. [14] employed large memory storage and retrieval (LAMSTAR) artificial neural networks to predict apnea and hypopnea using six physiological signals obtained from a set of polysomnography studies. The prediction performed best using 30 s segments to predict events up to 30 s into the future. The study in Taghizadegan et al. [15] used common single signals (EEG, ECG, and respiration) to represent the dynamic behavior of the signals before and during OSA events. ResNet-18 and ShuffleNet were implemented as classifiers and the classification results obtained from different signals are fused using the weighted majority voting method. However, measuring all these signals is obtrusive and inconvenient for the patient. The study in Zhang et al. [16] developed an autonomous system to detect and predict respiratory events during sleep using a covert bed-integrated radio-frequency sensor. The system can retrieve continuous respiratory waveforms without the user’s awareness and feed them into a random forest machine learning model for disorder detection and prediction. However, their study was conducted on 27 participants, excluding individuals diagnosed with severe OSA.

We developed a sleep event prediction framework based on low-frequency breathing-induced vibrations obtained from contactless piezoelectric sensors. According to the scoring criteria of sleep apnea, the respiratory signals are most directly related to respiratory dynamics. Since the breathing vibration signal contains body movement information and similar information with the respiratory signals, it is promising to provide enough information for the event prediction task. Physiological signals are inherently time-varying and sensitive to noise, making accurate feature extraction crucial for optimal classification performance. Deep learning methods enable the automatic extraction of features from data, facilitating their application in time series analysis [13,17,18]. Deep learning methods that have been successfully applied to sleep data analysis include recurrent neural networks (RNNs) [19,20,21], convolutional neural networks (CNNs) [22,23], transformers, and their combinations [24,25,26]. Our method leverages a combination of a CNN and a transformer to effectively capture local and global features in the respiratory vibration signals for accurate prediction. We evaluate our model on a clinical dataset of 105 subjects and demonstrate its effectiveness in the prediction of respiratory events. Our method shows promise for improving the clinical management of OSA.

The main contributions of our work are the following:

- A novel contactless scheme based on deep learning and breathing vibration signals is developed for sleep apnea event prediction. Our method can effectively predict respiratory events without disturbing the sleep of the subjects.
- A novel CNN–transformer network is proposed for prediction. It leverages the advantages of both CNN and transformer architectures, to effectively capture both local and global features present in the respiratory signals for prediction.
- The proposed method is validated on a dataset of 105 subjects from a public hospital and obtained a prediction accuracy of 85.9%. The method outperformed classical time series classification methods in terms of accuracy, sensitivity, and F1 score, demonstrating its effectiveness for the prediction of sleep apnea events. Two types of cross-validation were performed to demonstrate the generalization of our model.

## 2. Materials and Methods

### 2.1. Data Collection and Preparation

Our model was trained on a dataset collected from the Beijing Anzhen Hospital, which includes 105 participants (87 males and 18 females). The Ethics Committee of Beijing Anzhen Hospital approved this study. Patients who met any of the following criteria were excluded: (1) previously diagnosed with OSA, currently receiving CPAP therapy, or undergoing oxygen therapy; (2) disabled patients with heart failure or stroke. The detailed demographic information is shown in Table 1. The severity of OSA is determined by AHI, defined as the average number of respiratory events per hour of sleep. AHI scores of less than 5 indicate a normal condition, while scores between 5 and 15 are considered mild OSA. If the AHI falls between 15 and 30, it is classified as moderate OSA, and scores exceeding 30 indicate severe OSA.

**Figure 1:**
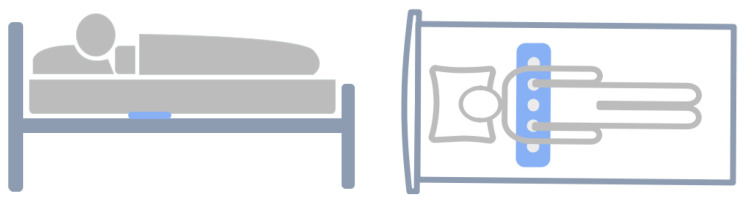
Illustration of the sleep data recording procedure, where the blue represents the sensors. They are located between the mattress and the bed frame, not in contact with the human body, and positioned near the chest.

As illustrated in Figure 1, Breathing activity is recorded by five piezoelectric sensors placed in rows under the mattress. These sensors measure body recoil micro-movements caused by respiration at a sampling rate of 5 Hz. Each participant took a home sleep apnea test (HSAT) for one night while the micro-movements were recorded simultaneously. HSAT was carried out using Alice PDx (Amsterdam, The Netherlands) [27], which compromises several channels with attached sensors that record oxygen saturation (SpO2), ECG, nasal airflow, respiratory effort, and body position. Recordings with missing respiratory signals due to sensor detachment or misplacement or those with a recording time less than 4 h were excluded from this study. The non-contact respiratory signals and HSAT signals were synchronized based on signal correlation. The HSAT signals was labeled by a sleep center specialist according to the American Academy of Sleep Medicine (AASM) manual [4].

**Figure 2:**
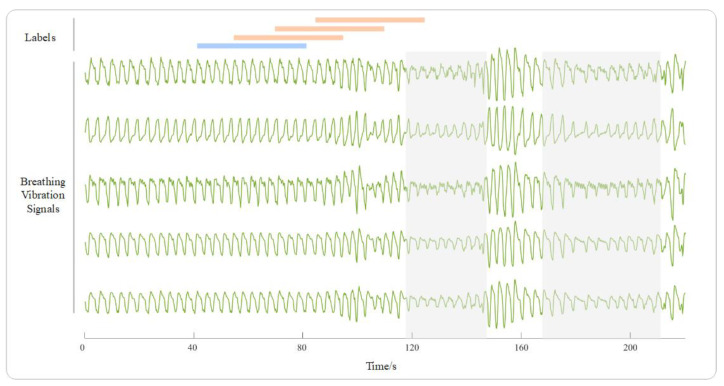
Sample data depicting: the green line in the figure represents the 5-channel respiratory vibration signals, the shaded area represents the occurrence of respiratory events, and the blue and orange lines, respectively, represent labels of “normal” and “prior”.

The recordings were segmented into epochs of 40 s with a sliding step of 15 s. We first divided the epochs into two types: respiratory events and normal breathing. If an apnea or hypopnea event occurs for more than 16 s within an epoch, the epoch is defined as a disordered epoch; otherwise, it is considered a normal breathing epoch. Epochs under off-bed conditions and segments affected by artifacts are excluded. We define the three normal breathing epochs before a respiratory event as “prior.” By distinguishing them from other normal breathing epochs, we can predict respiratory events. An example of epoch annotation is shown in Figure 2. Since the number of normal epochs is 5.2 times that of prior epochs, we randomly select the same number of epochs as the latter from the former to form a dataset, with a total of 38,985 epochs.

### 2.2. Analysis Model

**Figure 3:**
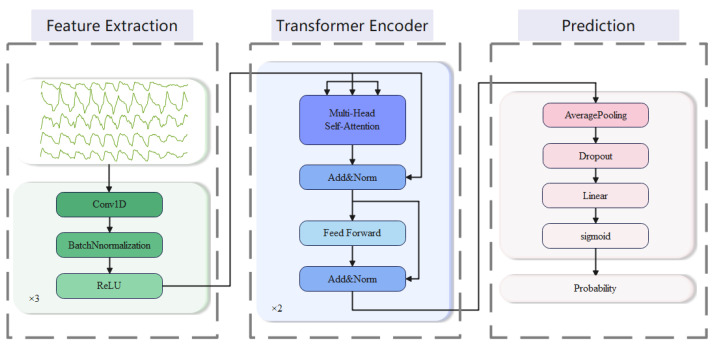
Diagram of the respiratory events prediction architecture.

We employ a CNN–transformer network for predicting respiratory events. The prediction architecture is shown in Figure 3. Firstly, we input the respiratory signal into the CNN module for high-dimensional feature learning and dimensionality conversion. Then, we feed the features into the transformer module for sequence modeling and capturing long-range dependencies. Finally, we perform average pooling on the output of the transformer module and feed it into a fully connected layer to perform binary classification, predicting whether the current segment is a precursor to a respiratory event. The following are the details of the model:

1. Feature extraction: We employ three 1D convolutional blocks to extract features. Each of the blocks consists of three sub-layers, which perform in turn: 1D-CNN layer, batch normalization (BN) layer, and ReLU activation layer. The first block has a convolutional kernel size of 3, while the next two blocks have convolutional kernel sizes of 29. The number of output channels is set to 64, and the padding is 1 in the first block and 14 in the last two blocks. The smaller kernel size captures local features with a smaller receptive field, while the larger kernel size captures global features with a larger receptive field. By combining them, the model can capture both local and global features, leading to a more comprehensive representation of the input signals.
2. Transformer encoder: We employ a stack of 2 transformer encoders to encode the high-dimensional features output. These encoded representations can effectively capture long-range dependencies in the sequence, providing strong support for subsequent classification tasks. Each transformer encoder consists of a multi-head self-attention layer and a position-wise feed-forward layer (FFN) [28].

The multi-head attention mechanism allows for the parallel computation of multiple attention heads, which can focus on different subspaces of information, capturing different attentional features. This helps to reduce noise and uncertainty in individual attention heads and improve the robustness of attention. The multi-head self-attention layer is calculated as

(1) $$MultiHead\left(Q,K,V\right)=Concat\left({Head}_{1},\ldots ,{Head}_{h}\right)W^{o}$$

where $W^{o}$ denotes the multi-headed trainable parameter weights, and ${Head}_{i}$ refers to the *i*-th attention head. The latter is calculated as

(2) $${Head}_{i}=Attention\left(QW_{i}^{Q},KW_{i}^{K},VW_{i}^{V}\right)$$

where *Q*, *K*, and *V* are the query, key, and value matrices, respectively, and $W_{i}^{Q},W_{i}^{K},W_{i}^{V}$ are the projection matrices for the *i*-th attention head. The attention score for input features is computed as the dot product of their respective query, key, and value vectors, which are obtained by linearly transforming the input features. The function can be expressed as

(3) $$Attention\left(Q,K,V\right)=softmax\left(\frac{QK^{T}}{\sqrt{d_{k}}}\right)V$$

where $\frac{1}{\sqrt{d_{k}}}$ is the scaling factor. The self-attention mechanism allows each position in the input sequence to be computed as a weighted average of the other positions, thus modeling dependencies between different parts of the sequence. The FFN layer consists of two linear layers with a ReLU activation function in between. It applies a non-linear transformation to enhance the model’s representational power. The FFN is calculated as

(4) $$FFN\left(x\right)=max\left(0,xW_{1}+b_{1}\right)W_{2}+b_{2}$$

After experiments, we set the number of attention heads in each self-attention mechanism to 8. In the FFN, we set a hidden layer with a middle dimension of 128 and use the ReLU activation function. For the input and output dimensions, we set them to 64.

3. Prediction: After the output of a transformer encoder, an average pooling layer and a dropout layer are typically applied. We use the average pooling layer to reduce the dimensionality of the output. A dropout layer is used to prevent overfitting with a parameter set to 0.5. Then, the result is mapped to the target output dimension through a linear layer and finally mapped to between 0 and 1 through a sigmoid layer to obtain the output probability.

Table 2 summarizes the parameters of the layers in the proposed model. In this table, “d_model” denotes the embedding output size, “nhead” represents the number of attention heads, “dim_feedforward” indicates the dimension of the hidden layer, and “num_layers” specifies the number of stacked transformer encoders.

### 2.3. Model Evaluation

We adopt commonly used metrics to assess the performance of binary classification, which include accuracy, sensitivity, and F1-score. The sensitivity metric is an important indicator in the field of biomedical research. In our task, the sensitivity metric can help us evaluate the ability of the model to identify real respiratory event precursors. F1-score provides a single value to reflect the overall performance of the model. The details are as follows:

(5) $$Accuracy=\frac{TP+TN}{TP+FP+FP+FN}$$

(6) $$Sensitivity=\frac{TP}{TP+FN}$$

(7) $$F1=\frac{2\times TP}{2\times TP+FP+FN}$$

## 3. Experiments and Results

### 3.1. Experiment Details

We adopted two manners to train and test our model. First, we employed the k-fold cross-validations (CV) to test the skill of the model on new data. We divided the whole dataset into a separate training set (70%), validation set (10%), and test set (20%), and the process was repeated five times until all cases had been tested as unseen data. Then, we employed the leave-one-out (LOO) CV to test the skill of the model on data from new subjects. We performed stratified sampling according to the severity of OSA cases and divided the dataset into five groups as shown in Table 3. The process was repeated five times until all subjects had been tested. Stratified sampling according to the severity of OSA cases helps to ensure that each group is represented fairly in the evaluation process.

To validate the effectiveness of our proposed model, we conducted ablation and comparative experiments on our clinical dataset. Firstly, we designed ablation experiments to evaluate the impact of the CNN and transformer modules on the performance. Additionally, since there are few studies related to respiratory prediction, we compared the CNN–transformer model with other commonly used deep learning models for time series data processing and classification tasks, including GRU, LSTM, BiLSTM, and their combinations with a CNN. These comparisons were performed to verify the effectiveness and superiority of our proposed model.

The experiments were performed on a computer with 1 CPU at 2.6 GHz, 1 NVIDIA GeForce RTX2060 GPU, and 64 GB memory. The proposed model was developed using Pytorch [29]. In the model, we used the adaptive moment estimation (Adam) optimizer with default parameters and a learning rate of 1 × 10^−4^ [30]. Binary cross entropy loss was used as the loss function.

### 3.2. Ablation Study

To verify the effectiveness of the CNN and transformer modules, a set of ablation experiments were conducted in this study with the same experiment setup. The CNN refers to the modified proposed model with the transformer blocks removed. The transformer refers to the modified proposed model with the CNN blocks removed. A 1D convolution with a kernel size of 1 was employed to realize the dimensionality conversion instead of the original CNN module.

The results are listed in Table 4. It is noted that the proposed model outperforms the other two models. The CNN–transformer model achieved an overall accuracy of 85.9% in the five-fold CV, which demonstrated the effectiveness of the proposed model for the classification of respiratory signals. There was a clear decrease in the model’s ability to identify the prior cases, with the F1-score dropping by 8.8% and 5.9% on the five-fold CV and the LOO CV, respectively, when the transformer module was removed. When the CNN module was removed, there was a decrease in the accuracy, sensitivity, and F1 score metrics by 5.6%, 3.5%, and 5.3%, respectively, on the five-fold CV and by 2.3%, 2.8%, and 2.6%, respectively, on the LOO CV.

### 3.3. Performance Comparison

We compared multiple commonly used deep learning time series data classification models, including GRU, LSTM, BiLSTM, and their combinations with a CNN. Specifically, the CNN-GRU, CNN-LSTM, and CNN-BiLSTM models were created by replacing the transformer blocks with GRU, LSTM, and BiLSTM blocks, respectively. The GRU, LSTM, and BiLSTM models refer to the CNN-GRU, CNN-LSTM, and CNN-BiLSTM models with the CNN blocks removed. For all these models, the number of hidden layer features is set to the same as it in the transformer encoder, i.e., 128. Additionally, the number of layers is also the same, i.e., 2.

As demonstrated in Table 5, the proposed model outperforms the hybrid models combining a CNN and different RNNs, and the RNN models perform the worst. The hybrid models combined of a CNN and different RNNs achieved similar performances. In the five-fold CV, BiLSTM achieved better performance than the CNN-RNN models. However, in the LOO CV, the performance of BiLSTM was significantly worse than that of the CNN-RNN models. Among the compared models, CNN-BiLSTM achieved the best performance. Our proposed model showed significant improvements over CNN-BiLSTM in both five-fold cross-validation and LOO validation. Regarding the F1 score, our model achieved increases of 5.3% and 3.8%, while in terms of sensitivity, the improvements were 11.3% and 7.4%.

**Figure 4:**
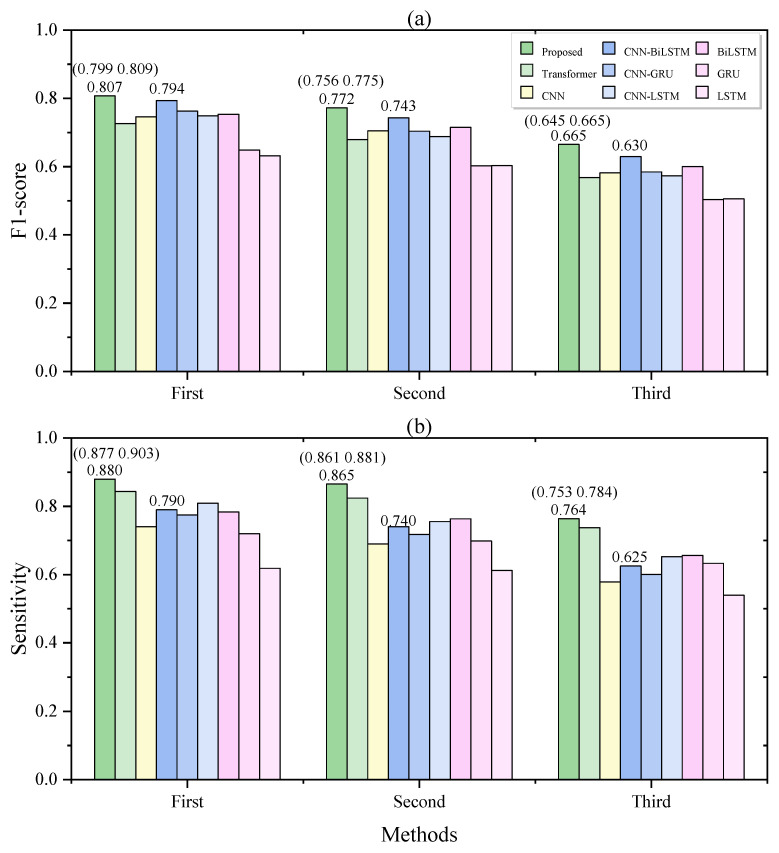
The F1-scores (**a**) and sensitivities (**b**) of different methods in detecting the first, second, and third segments before the respiratory events. The values in parentheses represent 95% confidence intervals.

We further analyzed the prediction performance of the first, second, and third segments preceding sleep apnea events. Figure 4 demonstrates that our model achieved the best performance in detecting “prior” segments at three different time intervals. The “first” segment represents the closest segment to the apnea event, while the “third” segment corresponds to the furthest segment preceding the apnea event. Sensitivity represents the detection rate of the segments, and our proposed model achieved detection rates of 88.0%, 86.5%, and 76.4% for the three different time intervals. This performance is higher than that of all other models. Our model achieved a detection rate of 76.4% for the earliest precursor segments, which is 13.9% higher than that of the CNN-BiLSTM model.

**Figure 5:**
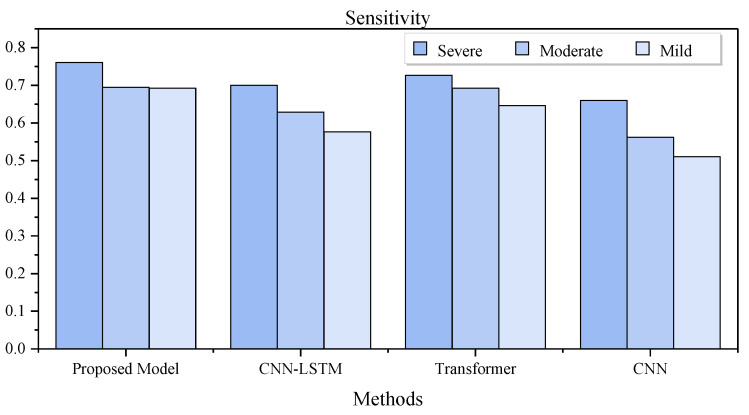
Sensitivity of different methods in different types of severity cases.

Table 6 lists the detection performance of methods in different severity levels of the OSA patients in LOO CV. As can be seen, our method achieved the best results in all severity groups, especially in the subjects with severe OSA. Figure 5 illustrates that our method maintains a good detection rate for the different severity levels, while the detection rates of other methods gradually decrease as the severity decreased.

## 4. Discussion

In this study, we aim to develop a novel unobtrusive framework for predicting breathing events to improve OSA treatment schemes and clinical management. We proposed a CNN–transformer model to classify normal breathing and breathing event precursors. We evaluated our framework on a clinical dataset of 105 subjects with different types of OSA severities in both five-fold CV and LOO CV. We achieved an accuracy of 85.9%, a sensitivity of 85.8%, and a F1 score of 84.7%, which is better than other common time series classical models.

The results in Table 4 illustrated that combining a CNN and a transformer is advantageous for the prediction of OSA events. CNNs are good at capturing local patterns for modeling short-term dependencies, but they cannot learn long-term dependencies due to the limited receptive field. The CNN model performs much worse than the models with transformer blocks in identifying prior segments. Transformers, on the other hand, are capable of learning global contexts and long-term dependencies. By combining a CNN and a transformer, the proposed method can effectively model both short-term and long-term dependencies within respiratory signals. The addition of CNN modules with different kernel sizes improved the overall performance of the proposed model. Good predictive performance in the OSA population is meaningful as it can help optimize the clinical management of OSA patients. Table 6 further confirms the effectiveness of our model, as our model outperforms individual modules and the classical model in predicting respiratory events in OSA patients across different severity levels. This suggests that our method has the potential to be applied in clinical settings to optimize the treatment of OSA patients.

Our framework has several advantages. First, our method does not interfere with subjects’ sleep or treatment, and the signal acquisition devices are suitable for both hospital and home environments. Secondly, the proposed CNN–transformer model achieves better performance compared to common methods. The CNN–transformer model utilizes self-attention mechanisms to achieve global interaction between any two positions without relying on hidden states to pass information. Apart from this, it utilizes multi-head attention mechanisms to achieve parallel computation in multiple subspaces, thereby capturing features of different levels of the temporal signal. In contrast, RNN models typically only capture features of a single dimension or aspect of the temporal signal. Finally, we performed two modes of validation on a real clinical dataset, including five-fold CV and LOO CV, demonstrating the effectiveness and robustness of our framework for predicting respiratory events. Our model achieved high accuracy and robustness in predicting non-intrusive respiratory events, with an average accuracy of 85.6% using five-fold CV and 75.4% using LOO CV. These results indicate that our method can effectively predict respiratory events in real clinical situations, promising to provide strategies for the treatment and management of OSA.

Table 7 summarizes the inter-subject and intra-subject results of the proposed system and the previous events prediction studies that used respiratory-related signals. The proposed system demonstrated better per-segment prediction results. Taghizadegan et al. [15] computed the recurrence plot (RP) of the signals by selecting appropriate parameters and focused on distinguishing signals before and during the occurrence of OSA events. Our proposed method achieved slightly inferior results in inter-subject results. Zhang et al. [16] extracted 37 features for the prediction of respiratory events, and the proposed method does not require manual feature extraction. These two studies involved 16 and 27 subjects, respectively, with 12 out of the 16 subjects in Taghizadegan et al. [15] having severe OSA, while Zhang et al. [16] did not include subjects with severe OSA. In contrast, our study included 105 subjects with varying degrees of severity, providing a more comprehensive validation of our model.

Further improvements can be made in our study. Our dataset includes 105 whole-night recordings from 87 male patients and 18 female patients. To improve our study, more female subjects could be included in our dataset to achieve a more balanced representation of both genders.

## 5. Conclusions

We developed a novel deep-learning-based framework for unobtrusive breathing event prediction. We proposed a novel CNN–transformer model for respiratory event prediction, which proved to be effective on a clinical dataset of 105 subjects. Our model combined the strengths of CNN and transformer architectures, allowing it to capture both local and global features of the input signals. Via extensive evaluations, we demonstrated that our model outperformed several classical models, including the RNN-based and CNN-RNN-based models, in terms of accuracy, sensitivity, and F1 score metrics. We believe that our framework can provide a new perspective for improving OSA treatment modes and clinical management.

## Figures and Tables

**Table 1:** Demographics of the participants.

Participants (#) (male)	105 (87)
Age (years)	51.0 ± 13.1
BMI (kg/m^2^)	28.7 ± 4.7
AHI (events/h)	21.9 ± 18.8
Normal/mild/moderate/severe OSA cases (#)	17/35/20/33

BMI: Body Mass Index; AHI: Apnea and Hypopnea Index; OSA: Obstructive Sleep Apnea.

**Table 2:** The parameters of the proposed model.

Module	Layer	Output Size	Parameters
Feature Extraction	Convolutional block	32 × 200	Kernel size: 3, stride: 1, padding: 1
	Convolutional block	32 × 200	Kernel size: 29, stride: 1, padding: 14
	Convolutional block	64 × 200	Kernel size: 29, stride: 1, padding: 14
Transformer Encoder	Transformer	200 × 64	d_model: 64, nhead: 8 dim_feedforward: 128 dropout: 0.3, num_layers: 2
Prediction	Average Pooling	64	Kernel size: 200
	Dropout	64	p: 0.5
	Linear	1	

**Table 3:** Five groups in the leave-one-out CV.

	Fold 1	Fold 2	Fold 3	Fold 4	Fold 5	Total
Normal cases	3	3	4	4	3	17
Mild cases	7	7	7	7	7	35
Moderate cases	4	4	4	4	4	20
Severe cases	7	7	6	6	7	33
Total	21	21	21	21	21	105

**Table 4:** Results of the ablation study.

	Five-fold CV			LOO CV		
	Accuracy	Sensitivity	F1	Accuracy	Sensitivity	F1
CNN	0.798	0.685	0.770	0.715	0.600	0.678
Transformer	0.803	0.812	0.805	0.718	0.698	0.711
Proposed	0.859	0.847	0.858	0.741	0.726	0.737

**Table 5:** Performance comparison of different time series classification models.

	Five-Fold CV			LOO CV		
	Accuracy	Sensitivity	F1	Accuracy	Sensitivity	F1
GRU	0.734	0.692	0.724	0.569	0.583	0.575
LSTM	0.728	0.597	0.685	0.572	0.586	0.578
BiLSTM	0.809	0.746	0.797	0.622	0.600	0.614
CNN-GRU	0.801	0.714	0.783	0.723	0.605	0.686
CNN-LSTM	0.801	0.754	0.791	0.720	0.633	0.694
CNN-BiLSTM	0.824	0.734	0.805	0.719	0.652	0.699
Proposed (95%CI ^1^)	0.859 (0.856 0.860)	0.847 (0.843 0.867)	0.858 (0.856 0.859)	0.741 (0.736 0.743)	0.726 (0.718 0.737)	0.737 (0.735 0.738)

^1^ CI: Confidence interval.

**Table 6:** Performance of methods in different types of severity cases.

	Accuracy			Sensitivity			F1-Score		
	Severe	Moderate	Mild	Severe	Moderate	Mild	Severe	Moderate	Mild
CNN	0.677	0.678	0.734	0.660	0.562	0.510	0.722	0.655	0.544
Transformer	0.707	0.708	0.723	0.727	0.692	0.646	0.763	0.719	0.598
CNN-BiLSTM	0.700	0.695	0.730	0.700	0.629	0.577	0.756	0.696	0.575
Proposed	0.738	0.716	0.743	0.761	0.694	0.692	0.794	0.730	0.636

**Table 7:** Comparison results of sleep apnea event prediction studies using respiratory-related signals.

Study	No. of Subjects	Sensor Type	Method	Per-Segment		Per-Subject	
				Sen	Acc	Sen	Acc
[15]	16	Respiratory belts	ShuffleNet	0.803	0.808	0.766	0.767
[16]	27	Radio-frequency sensors	Random Forest	0.746	0.819	0.727	0.817
Proposed	105	Piezoelectric sensors.	CNN–Transformer	0.847	0.859	0.726	0.741

Acc: Accuracy; Sen: sensitivity.

## Data Availability

The data are not publicly available.
